# Natural Killer Cell-Derived Extracellular Vesicles Exhibit Cytotoxicity Against Bulk Tumor Cells and Cancer Stem Cells in Triple-Negative Breast Cancer

**DOI:** 10.3390/nano16090525

**Published:** 2026-04-27

**Authors:** Melanie Kirkby, Frederic St-Denis-Bissonnette, Marena D. Diab, Karan Mediratta, Anna Korobkow, James Humber, Peter Han, Gauri Muradia, Michele Ardolino, Seung-Hwan Lee, Derrick J. Gibbings, Dylan Burger, Lisheng Wang, Jessie R. Lavoie

**Affiliations:** 1 Biologic and Radiopharmaceutical Drugs Directorate, Health Canada, Ottawa, ON K1A 0K9, Canada; mkirk071@uottawa.ca (M.K.); fstde005@uottawa.ca (F.S.-D.-B.); akoro009@uottawa.ca (A.K.); gauri.muradia@hc-sc.gc.ca (G.M.); 2 Department of Biochemistry, Immunology and Microbiology, University of Ottawa, Ottawa, ON K1H 8M5, Canada; mdiab042@uottawa.ca (M.D.D.); kmedi072@uottawa.ca (K.M.); jhumb080@uottawa.ca (J.H.); zhan100@uottawa.ca (P.H.); m.ardolino@uottawa.ca (M.A.); seunglee@uottawa.ca (S.-H.L.); 3 Centre for Infection, Immunity and Inflammation, University of Ottawa, Ottawa, ON K1H 8M5, Canada; 4 Cancer Therapeutics Program, Ottawa Hospital Research Institute, Ottawa, ON K1H 8L6, Canada; 5 Department of Cellular and Molecular Medicine, University of Ottawa, Ottawa, ON K1H 8M5, Canada; gibbings@uottawa.ca (D.J.G.); dburger@uottawa.ca (D.B.); 6 Kidney Research Centre, Ottawa Hospital Research Institute, Ottawa, ON K1H 8L6, Canada; 7 Regenerative Medicine Program, Ottawa Hospital Research Institute, Ottawa, ON K1H 8L6, Canada

**Keywords:** cancer stem cells (CSCs), natural killer cell-derived extracellular vesicles (NK-EVs), patient-derived xenograft (PDX), solid tumor, triple-negative breast cancer (TNBC)

## Abstract

Triple-negative breast cancer (TNBC) remains a significant challenge in oncology, contributing to a significant portion of cancer-related deaths among women. Current therapeutic options, including chemotherapy, surgery, radiation, and hormonal targeting therapies, exhibit limited efficacy, necessitating the exploration of innovative treatment modalities. The emergence of drug resistance and the persistence of cancer stem cells (CSCs) further emphasize the urgent need for novel therapeutic strategies. In this context, natural killer cell-derived extracellular vesicles (NK-EVs) have emerged as a promising cell-free therapeutic approach that exhibits high tumor infiltration and cytotoxicity against cancer cells and CSCs. This study aims to investigate the efficacy of NK-EVs as a therapeutic strategy for TNBC using various clinically relevant models, including patient-derived xenografts. Pathway analysis suggests strong activation of apoptosis via canonical caspase activation, as well as necrosis, thereby confirming the important cytotoxic effect of NK-EVs. Interestingly, NK-EVs were also found to suppress TNBC CSCs by disrupting their functionality and viability, and NK-EV treatment increased the expression of apoptosis markers in both CSCs and non-CSCs. By elucidating the therapeutic efficacy and translational potential of NK-EV-based interventions in TNBC, these findings offer critical insights for the development of future immunotherapeutic strategies against this aggressive subtype of breast cancer.

## 1. Introduction

Triple-negative breast cancer (TNBC) represents one of the most aggressive subtypes of breast malignancies, characterized by the absence of estrogen receptors, progesterone receptors, and human epidermal growth factor receptor 2 (HER2). Although TNBC accounts for approximately 14% of all breast cancer cases, it is responsible for a disproportionately high number of cancer-related deaths among women in North America [1,2]. Currently, the five-year survival rate for TNBC patients is approximately 77%, markedly lower than the ~90% observed across all breast cancer subtypes.

The mainstay treatment program for TNBC—a combination of chemotherapy, surgery and radiation therapy—often fails to achieve durable responses due to intrinsic or acquired resistance mechanisms, leading to poor prognosis and high relapse rates [3]. This inherent resistance underscores the urgent need for innovative treatment modalities that can circumvent resistance pathways and effectively address the aggressive nature of TNBC. Furthermore, currently approved hormonal and HER2-targeted therapies are ineffective in this subtype, as TNBC lacks the corresponding molecular targets [4].

Another major consequence of chemotherapy is the enrichment of cancer stem cells (CSC) following treatment [5,6,7]. Although CSCs are a minor subset of the tumor population, they possess extensive self-renewal and differentiation capacities. Residual CSCs can reconstitute the entire tumor, driving recurrence and metastatic progression [8,9,10]. Their pronounced chemoresistance, mediated by enhanced drug efflux and phenotypic plasticity through the reversible epithelial–mesenchymal transition, renders conventional TNBC treatments largely ineffective [8,10,11,12,13,14]. Furthermore, CSC survival and tumorigenesis are sustained through the activation of key signaling pathways, particularly the Hippo-YAP signaling axis, which plays a central role in CSC maintenance, tumorigenesis, and metastasis [14,15,16]. Current research suggests that inhibition of this pathway suppresses CSC viability and impedes tumor progression [17,18]. Thus, there is an urgent need for novel TNBC therapeutics capable of targeting both bulk tumor cells and the CSC compartment to achieve durable remission.

The field of extracellular vesicle (EV)-based biotherapeutics for cancer treatment is rapidly advancing worldwide [19,20,21]. EVs are nanosized membrane-bound vesicles released by virtually all cell types and serve as potent mediators of intercellular communication. Among these, EVs derived from immune cells, particularly natural killer (NK) cells, have garnered considerable attention due to their potential anti-tumor and immunomodulatory properties [22,23]. NK cells are a crucial component of the innate immune system, capable of recognizing and eliminating tumor cells without prior sensitization. Complementing their direct cytolytic activity, NK cell-derived EVs (NK-EVs) have shown potent anti-tumor effects in preclinical models by inducing apoptosis, inhibiting cancer cell proliferation, and enhancing immune surveillance [21,23,24,25,26,27,28,29]. The NK-EVs’ complex, bioactive cargo—which includes proteins (e.g., perforin, granzyme A/B, granulysin), cytokines (e.g., IFN-γ, IL-6, IL-10), and miRNA (e.g., miR-21, miR-155, miR-181)—is directly responsible for facilitating the NK-EVs’ cytolytic functioning [26,27,28,29,30]. Recently, our group demonstrated the importance of the biomolecular corona in influencing NK-EV behavior, cytokine release, and subsequent viability, providing valuable insight into the NK-EVs’ mode-of-action (MoA) [30]. Furthermore, NK-EVs have also demonstrated efficacy against treatment-resistant leukemic cells with a CSC-like phenotype, suggesting their potential role in preventing tumor recurrence [3]. Despite their cytotoxic payload, various studies, including our group’s recent investigation in healthy human fibroblasts, have demonstrated that NK-EVs do not compromise the viability of healthy, non-malignant cells [26,27,31,32]. This suggests that NK-EVs may preferentially target cancer cells, recapitulating the specificity of their parental NK cells and supporting their potential as a therapeutic and drug delivery platform.

Although promising, most preclinical studies investigating NK-EVs to date have been constrained by the limited clinical relevance of disease models and non-physiological EV dosing regimens, underscoring the need for translationally optimized approaches to evaluate NK-EV efficacy in clinically meaningful settings. Here, this study investigates NK-EVs as a therapeutic strategy for treating TNBC using clinically relevant models. NK-EVs were produced through a clinically relevant, GMP (Good Manufacturing Practices)-compliant, large-scale biomanufacturing workflow from NK92-MI cells. The detailed workflow, as well as the characterization and safety profile of the resulting NK-EV products, were described in our previous publication [26]. To assess their translational potential in TNBC, NK-EVs were evaluated in clinically relevant models, including patient-derived xenografts (PDX), which closely recapitulated the pathological features and the clinical behaviors of TNBC, offering a physiologically relevant platform for preclinical assessment [33]. NK-EVs exhibited a rapid, dose-dependent cytotoxic effect against TNBC cells and PDX models, as evidenced by increased apoptosis, necrosis, and caspase activation. Furthermore, NK-EV treatment reduced the viability of TNBC CSCs, suggesting an impact on tumor recurrence. Collectively, the findings of this work support the clinical translation of NK-EVs as a novel and promising therapeutic strategy to address the unmet needs of TNBC patients.

## 2. Materials and Methods

### 2.1. Cell Culture Parameters

NK92-MI cells (purchased from ATCC, Manassas, VA, USA) were cultured in serum-free, xeno-free and feeder-free ImmunoCult™-XF T Cell Expansion Medium (StemCell Technologies, Vancouver, BC, Canada) and maintained within 3–8 × 10^5^ cells/mL [26]. Freestyle 293F cells (purchased from Thermo Fisher, Waltham, MA, USA) were cultured in FreeStyle™ 293 Expression Medium (Gibco, Waltham, MA, USA) on a shaker platform where the density was maintained between 1–3 × 10^6^ cells/mL. Breast cancer HCC70 cells (purchased from ATCC, Manassas, VA, USA) were cultured in RPMI-1640 with GlutaMAX™ (Gibco, Waltham, MA, USA) with 10% Heat Inactivated Fetal Bovine Serum (HI-FBS, Gibco, Waltham, MA, USA). Breast cancer MDA-MB-231 cells (purchased from ATCC; Manassas, VA, USA) and breast cancer MDA-MB-468 cells (purchased from ATCC, Manassas, VA, USA) were cultured in Hams F-12 Medium (Corning, Corning, NY, USA) and high-glucose DMEM with GlutaMAX™ (Gibco, Waltham, MA, USA) at a 1:1 ratio with 10% HI-FBS. Breast cancer SUM149PT cells (purchased from Asterand, Detroit, MI, USA) were cultured in Hams F-12 Medium with 10% HI-FBS, 10 mM HEPES (Gibco, Waltham, MA, USA), 5 µg/mL hR Insulin (Gibco, Waltham, MA, USA), and 1 µg/mL hydrocortisone (Sigma-Aldrich, St. Louis, MO, USA). Breast cancer BT-549 cells (purchased from ATCC, Manassas, VA, USA) were cultured in RPMI-1640 with 10% HI-FBS and 10 µg/mL hR Insulin (Gibco, Waltham, MA, USA). Breast cancer MCF7 cells (purchased from ATCC, Manassas, VA, USA) were cultured in EMEM Medium (ATCC, Manassas, VA, USA) with 10% HI-FBS and 10 µg/mL hR Insulin (Gibco, Waltham, MA, USA). All adherent cells were passaged using TrypLe select (Gibco, Waltham, MA, USA) after washing the cells with sterile Dulbecco’s phosphate-buffered saline without Ca^2+^/Mg^2+^ (DPBS^−/−^, Gibco, Waltham, MA, USA) and seeded at 2–4 × 10^4^ cells/cm^2^. Cell lines were cultured at 37 °C in a 5% CO_2_ incubator and passaged every 2–3 days, except the 293F cell line, which was cultured at 37 °C in an 8% CO_2_ incubator. Cell counts were performed on the Cellometer Auto 2000 Viability Counter (Nexcelom BioScience LLC, Lawrence, MA, USA) using the ViaStain Acridine Orange and Propidium Iodide (AO/PI) Staining Solution (ESBE Scientific, Markham, ON, Canada) with the immune cell AO/PI program (channel 1: 470/535 nm for 0.5 s of exposure and channel 2: 540/605 nm for 3 s of exposure). The PCR detection kit assessed all cell lines for mycoplasma contamination (Abcam, Cambridge, UK).

### 2.2. NK-EV Biomanufacturing from NK92-MI Cells

NK-EVs were generated from NK92-MI cells using a clinically relevant large-scale biomanufacturing workflow that adheres to Good Manufacturing Practices (GMP), as previously described by our lab [26]. Briefly, the biomanufacturing of NK92-MI cells and NK-EVs was performed using a closed-loop hollow fiber bioreactor to generate large quantities of clinical-grade NK-EVs. NK92-MI cells are expanded in the cartridge and harvested at the end of the production cycle. Of note, NK-EVs were generated using ImmunoCult™-XF T Cell Expansion Medium, with EV-rich conditioned medium (CM) collected once daily and stored at −80 °C until further processing.

### 2.3. NK-EV Isolation and Characterization

NK-EV isolation and characterization were previously described by our lab [34]. Briefly, the EV-rich CM was thawed, centrifuged, and incubated for 4 h at 37 °C with 50 U/mL Benzonase Nuclease (MilliporeSigma, Burlington, MA, USA) and 1.5 mM of MgCl_2_ (MilliporeSigma, Burlington, MA, USA) while moderately shaking for nucleic acid digestion. Size exclusion chromatography-based processing was then performed on the AKTA Fast Protein Liquid Chromatograph (MilliporeSigma, Burlington, MA, USA). The obtained solution was concentrated using a pre-equilibrated 10 kDa Centricon Plus-70 Centrifugal Filter—regenerated cellulose membrane (Millipore Sigma, Burlington, MA, USA). Purified NK-EV samples were then analyzed using the NanoSight NS300 (V3.4; Malvern Pananalytical, Malvern, UK). For capture settings, the camera level was set to 14, and the detection threshold to 15 for analysis. The obtained concentrations were used to calculate the proper dosage for all experiments described. Protein and dsDNA levels were quantified using the Qubit 4 Fluorometer (Invitrogen, Carlsbad, CA, USA) as per the manufacturer’s instructions.

### 2.4. In Vitro Potency Evaluation of NK-EV Against TNBC Models Using the PrestoBlue Viability Assay

As previously described, a validated potency assay was employed to evaluate NK-EVs’ cytotoxicity against various breast cancer models [35]. A week before the cytotoxic co-culture assay, the various target cells were cultured as per Section 2.1. Using a 96-well Flat Clear Bottom Black Polystyrene TC-treated Microplate (Corning, Corning, NY, USA), 1500 adherent cells were seeded per well using a pipette repeater in technical triplicates. Cells were maintained in Fluorobrite DMEM (Gibco, Waltham, MA, USA), where the final well volume was normalized to 150 µL. Adherent cells were allowed to settle into the plate for 18–24 h at 37 °C with 5% CO_2_ before adding effector EVs at various concentrations. Untreated cells, 1X of Triton-X 100 (Sigma, cat#T-9284) detergent-treated cells, 10 µM Paclitaxel (PTX; Caymanchem, Ann Arbor, MI, USA)-treated cells and 293F-EV-treated cells were used as controls. Notably, the dosing volume was limited to 10% of the total assay volume. Except for the time course experiment, the PrestoBlue™ HS Cell Viability Reagent Assay (Invitrogen, Carlsbad, CA, USA) was added to each well (1X) after 4 h of treatment, where plate incubation was performed at 37 °C in a 5% CO_2_ incubator and protected from light (60 min incubation). Fluorescence was measured (Ex/Em 560/590 ± 9 nm) with a Microplate Reader (BioTek Synergy H1 Multimode Reader; Gen5 software V3.14; Agilent, Santa Clara, CA, USA), temperature-stabilized at 37 °C to reduce temperature variation on fluorescence measurements. Before acquisition, the plate reader mixed the plate for 30 s at 350–500 RPM to homogenize the content in each well.

### 2.5. NK-EV Treatment of Pre-Treated TNBC Cell Models

A week before the cytotoxic co-culture assay, the target cells were cultured as per Section 2.1. Using a 6-well flat-bottom TC-treated plate, 5 × 10^5^ cells/mL were seeded per well and allowed to settle in 1 mL of media. Cells were treated with NK-EVs (5 × 10^10^ EVs/mL) and allowed to incubate at 37 °C in a 5% CO_2_ incubator. 24 h following treatment, the media was replaced to remove dead cells. Cells were allowed to grow to confluency, after which cells were re-treated with NK-EVs and assessed according to Section 2.4.

### 2.6. Plate-Based Evaluation of Cell Death Pathways Following NK-EV Treatment

A week before the cytotoxic co-culture assay, the various target cells were cultured as per Section 2.1. Using a 96-well Flat Clear Bottom Black Polystyrene TC-treated Microplates, 1500 adherent cells were seeded per well using a pipette repeater. Cells were maintained in Fluorobrite DMEM at 37 °C with 5% CO_2_, where the final well volume was normalized to 100 or 200 µL (assay dependent). Adherent cells were allowed to settle into the plate for 18–24 h before adding effector EVs at various concentrations: 1 × 10^9^ and 1 × 10^10^ EVs/mL. Untreated cells, 1× of Triton-X 100 detergent-treated cells, and 10 μM PTX-treated cells were used as controls. For continuous measurement, 1× RealTime-Glo™ Annexin V Apoptosis (luminescence) and Necrosis (fluorescence; Ex/Em 485/525 ± 9 nm) Assay (Promega, Madison, WI, USA) was used. Detailed caspase activity was measured after 90 min of treatment using the Cell Event Caspase 3/7 Green Detection Reagent (Invitrogen, Carlsbad, CA, USA), the Caspase-Glo^®^ 8 (luminescence) Assay Systems (Promega, Madison, WI, USA), where 60 µM MG-132 Inhibitor was used to reduce background, and the Caspase-Glo^®^ 9 (luminescence) Assay Systems (Promega, Madison, WI, USA), where 60 µM MG-132 Inhibitor was used to reduce background. Readings were made using a prewarmed Microplate Reader (BioTek Synergy H1 Multimode Reader; Gen5 software V3.14). Before acquisition, the plate reader mixed the plate for 30 s at 350 RPM to homogenize the content in each well.

### 2.7. Ex Vivo Potency Evaluation of NK-EV Against TNBC-PDX Organotypic Slice Cultures

HCI-001, HCI-002, HCI-010, and HCI-015 tumor fragments were obtained from and characterized by the University of Utah (Table S1) [36]. PDX fragments of 2 × 2 mm in size were incubated in 24-well plates (i.e., organotypic slice culture) and allowed to settle in the plate for 18–24 h before beginning treatment. Over a five-day period, PDX fragments were treated every 24 h with NK-EVs (1 × 10^11^ EVs/mL), 10 nM PTX, a combination of NK-EVs (1.0 × 10^11^ EVs/mL) and 10 nM PTX, or left untreated. Viability assessment was performed daily using the PrestoBlue™ HS Cell Viability Reagent Assay as detailed in Section 2.4 after 3 h of incubation. To evaluate caspase 3/7 activation, 2 × 2 mm PDX fragments were seeded in 24-well plates and allowed to settle for 18–24 h before beginning treatment. The caspase 3/7 detection assay was performed as per Section 2.6.

### 2.8. Tumorsphere Formation Assay

The procedure for the tumorsphere formation assay was performed as previously described, with some modifications [37]. A week before the cytotoxic co-culture assay, the various target cells were cultured as per Section 2.1. Totals of 1500 E-cadherin+ MDA-MB-231 or 2500 SUM149PT cells were seeded into a 96-well ultra-low attachment plate and maintained in 1:1 DMEM and Hams F-12 supplemented with 2% B-27™ Supplement (Gibco, Waltham, MA, USA), 1% sodium pyruvate (Gibco, Waltham, MA, USA), 1% penicillin/streptomycin (Cytiva, Marlborough, MA, USA), 20 ng/mL basic fibroblast growth factor (R&D Systems, Minneapolis, MN, USA), and 20 ng/mL epidermal growth factor (R&D Systems, Minneapolis, MN, USA). Effector EVs were added to the plate and allowed to co-incubate with their treatment for 72 h. Tumorsphere viability was determined using the PrestoBlue viability assay as described in the Section 2.4.

### 2.9. RT-qPCR

A week before treatment began, the MDA-MB-231 cells were cultured as per the Section 2.1. Briefly, 3 × 10^5^ cells were seeded into 6-well plates and allowed to settle in the plate for 18–24 h prior to treatment. Total RNA was extracted using the RNeasy Mini kit (Qiagen, Toronto, ON, Canada). cDNA was obtained from RNA using the iScript cDNA Synthesis Kit (Bio-Rad, Hercules, CA, USA) as per the manufacturer’s instructions. Gene expression was measured using the CFX Opus 96 Real-Time PCR System (V5.3.022.1030; Bio-Rad, Hercules, CA, USA) in a reaction mixture consisting of 50% SyBr Green Supermix (Bio-Rad, Hercules, CA, USA), 37.5% RNAse-free water (Qiagen, Toronto, ON, Canada), 5% forward and reverse primers (all primers were purchased from Eurofin Genomics, Louisville, KY, USA), and 2.5% cDNA. The RT-qPCR script followed was (1) 90 s at 95 °C, (2) 10 s at 95 °C, (3) 30 s at 60 °C with a plate reading, (4) 30 s at 72 °C, (5) return to step 2 and repeat 45× total. Specific primer sequences are listed in Table S2. The results were normalized to the GAPDH housekeeping gene, and the relative fold changes in gene expression were calculated using the ^2ΔΔ^CT method.

### 2.10. Live Cell Imaging

MDA-MB-231 cells were tagged with red fluorescent protein (RFP) by lentiviral transduction of Incucyte Nuclight Red (Sartorius, Gottingen, Germany), followed by puromycin (Tocris Bioscience, Bristol, UK) selection. A week before treatment began, RFP+ MDA-MB-231 cells were cultured as per Section 2.1 for the MDA-MB-231 cell line. Cells were dissociated, filtered through a 40 µM strainer (Bio Basic, Markham, ON, Canada), and resuspended in DPBS^−/−^ supplemented with 2% FBS and 2 mM EDTA. To reduce non-specific binding, anti-human IgG Fc (Thermo Fisher, Waltham, MA, USA) was added at 4 °C for 10 min. Cells were incubated with anti-human BV650-CD44 (BD Biosciences, cat#743665) and anti-human PE-CD24 (BD Biosciences, Missisauga, ON, Canada) according to the manufacturer’s instructions. Fluorescence-activated cell sorting (FACS) into the CD44^high^/CD24^low^ and non-CD44^high^/CD24^low^ cells was conducted using the SH800 Cell Sorter (Sony Biotechnology, San Jose, CA, USA). Sorted cells were seeded back and cultured for 24 h before live cell imaging began. A total of 1500 cells were seeded into the well of a TC-treated 96-well plate and incubated with green Annexin-V (Sartorius, Gottingen, Germany) for live cell imaging in the Sartorius Incucyte S3 for 96 h. Treatment occurred immediately after the first images were captured at the 0 h time point.

### 2.11. Statistical Analysis

Data were expressed as the means ± the standard deviation (SD) or the standard error of the mean (SEM). The data were normalized to the control group for relative comparison, as indicated in each figure legend. The numbers of experimental and technical replicates used are indicated in the figure legends. Statistical analyses were performed using GraphPad Prism version 7.0 (GraphPad Software Inc., LaJolla, CA, USA). Unless otherwise indicated, one-way or two-way ANOVA followed by post hoc tests (Tukey’s or Sidak’s multiple comparisons) were used to assess data distribution, where a *p*-value of <0.05 was considered statistically significant. Significance differences are marked with a single (*p* < 0.05), double (*p* < 0.01), triple (*p* < 0.001), or quadruple (*p* < 0.0001) asterisk.

## 3. Results

All NK-EVs used in this study were generated from the NK92-MI cell line using a scalable biomanufacturing workflow. Comprehensive characterization of these NK-EVs has been previously reported by our laboratory [26]. Briefly, transmission electron microscopy, nanoparticle tracking analysis, and antigen/cytokine profiling were employed to validate NK-EV morphology, concentration, size distribution, and functional properties.

### 3.1. NK-EVs Rapidly Induce Dose-Dependent Cytotoxicity Across Monolayer TNBC Cell Line Models

**Figure 1:**
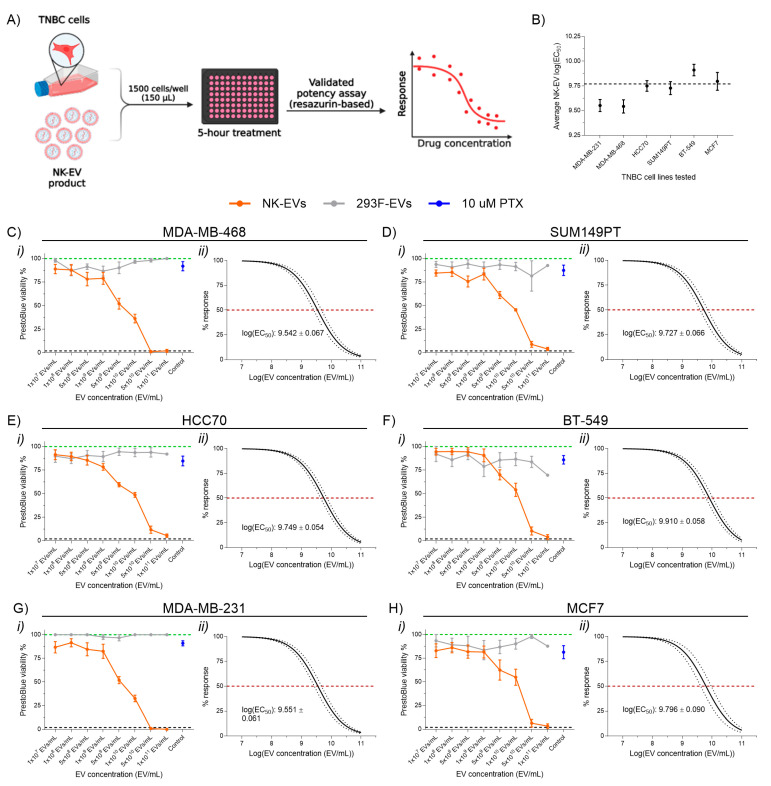
Cell viability of various TNBC cell lines following NK-EV treatment. (**A**) Schematic overview of experimental design. TNBC cells were treated for 5 h with various E:T ratios of NK-EVs (orange), 293F-EVs as a negative control (gray), or paclitaxel (10 µM PTX; blue). Cell viability was then assessed using an endpoint resazurin-based assay. This schematic was created with BioRender. (**B**) The average NK-EV EC_50_ value across all TNBC cell lines. Cell viability and EC50 analysis for individual cell line: (**C**) MDA-MB-468 cells, (**D**) SUM149PT cells, (**E**) HCC70 cells, (**F**) BT-549 cells, (**G**) MDA-MB-231 cells, and (**H**) MCF7 cells. Panels (**C**(**i**),**D**(**i**),**E**(**i**),**F**(**i**),**G**(**i**),**H**(**i**)) show RFU (green dashed line represents untreated cells, black dashed line indicates lysed cell control with Triton-X). Panels (**C**(**ii**),**D**(**ii**),**E**(**ii**),**F**(**ii**),**G**(**ii**),**H**(**ii**)) show EC50 curve fits for NK-EV treatment with 95% confidence and prediction intervals (red dashed line denotes 50% response). Data represent mean ± SEM from six independent experiments, each with technical triplicates.

To evaluate the cytotoxicity of NK-EVs on TNBC, a validated potency assay was performed using a panel of monolayer TNBC cell line models for 5 h following treatment (Figure 1A) [35]. The panel included basal-like cell lines (Basal-like 1: MDA-MB-468; Basal-like 2: SUM149PT, HCC70), a mesenchymal-like cell line (BT-549), a mesenchymal stem-like cell line (MDA-MB-231), and a luminal A breast cancer cell line (MCF7, ER+/PR+) as a non-TNBC comparator (Table S3). NK-EV treatment led to a significant and dose-dependent reduction in cell viability across all TNBC lines tested, with an average log(EC_50_) of 9.77 ± 0.14 EVs/mL (Figure 1B–G). In contrast, treatment with control 293F-EVs had no measurable effect on cell viability, confirming that the observed cytotoxicity was specific to NK-EVs rather than to the EV vehicle itself. Similarly, paclitaxel (PTX), an approved TNBC chemotherapeutic, did not induce significant cell death within the 5 h, consistent with its known requirement for at least 24 h to achieve a substantial cytotoxic effect [38,39]. This finding demonstrates the rapid and potent, dose-dependent and cytotoxic capacity of NK-EVs against TNBC cells. Notably, NK-EV treatment exhibited enhanced cytotoxicity against pre-treated TNBC cells (i.e., previously treated with NK-EVs at 5.0 × 10^10^ EVs/mL and then allowed to reach confluency) compared to their untreated counterparts at all NK-EV concentrations tested (Figure S1A). Pre-treated MDA-MB-231 cells achieved a log(EC_50_) of 9.25 ± 0.07 EVs/mL, compared to 9.56 ± 0.06 EVs/mL for untreated cells (Figure S1B,C). Similarly, the log(EC_50_) for pre-treated and untreated SUM149PT cells was 9.35 ± 0.05 EVs/mL and 9.73 ± 0.07 EVs/mL, respectively. Thus, NK-EV administration may sensitize cells for further rounds of treatment.

### 3.2. NK-EVs Exhibit a Transient, Time-Dependent Cytotoxicity in a Monolayer TNBC Model

**Figure 2:**
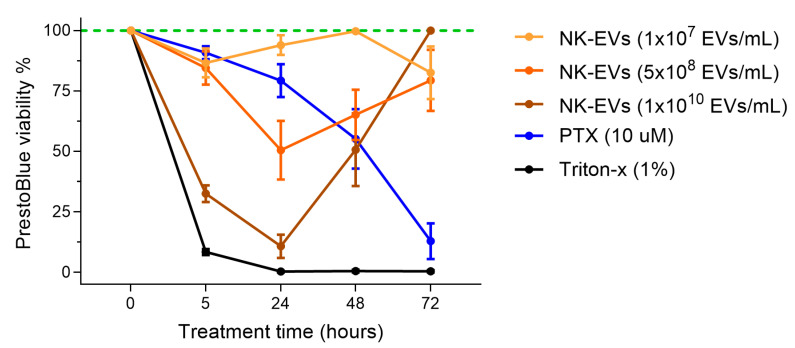
TNBC cell viability following NK-EV treatment over a 72 h time course. MDA-MB-231 cells were treated for 5, 24, 48 and 72 h with various NK-EV doses (1 × 10^7^ EVs/mL, yellow; 5 × 10^8^ EVs/mL, orange; and 1 × 10^10^ EVs/mL, brown) or with paclitaxel (10 μM PTX, blue). The results are presented as the normalized RFU values, with the green dashed line representing untreated control and black solid line representing lysed cell control (using Triton-X). Data represent mean ± SEM from six independent experiments, each with technical triplicates.

As NK-EV cytotoxicity was dose-dependent, we examined whether its cytotoxicity also varied over time. MDA-MB-231 TNBC cells were treated with NK-EVs for 5, 24, 48, and 72 h. At 24 h, the highest NK-EV concentration (1 × 10^10^ EVs/mL) significantly reduced cell viability to 10.78% ± 11.85% (Figure 2 and Figure S2). However, cell viability partially recovered to 50.72% ± 36.81% after 48 h and fully returned to baseline (100.00% ± 0.00%) by 72 h. Lower NK-EV doses showed a similar trend, reinforcing that NK-EV-induced cytotoxicity is transient and dose-dependent. Conversely, PTX did not significantly affect viability at 5 h but progressively reduced cell survival over time, with only 12.90% ± 18.07% of cells remaining viable after 72 h. These results suggest that NK-EVs induce rapid, short-lived cytotoxicity without sustained antiproliferative effects on cancer cells.

### 3.3. NK-EVs Activate Both Apoptotic and Necrotic Pathways in TNBC Cells

**Figure 3:**
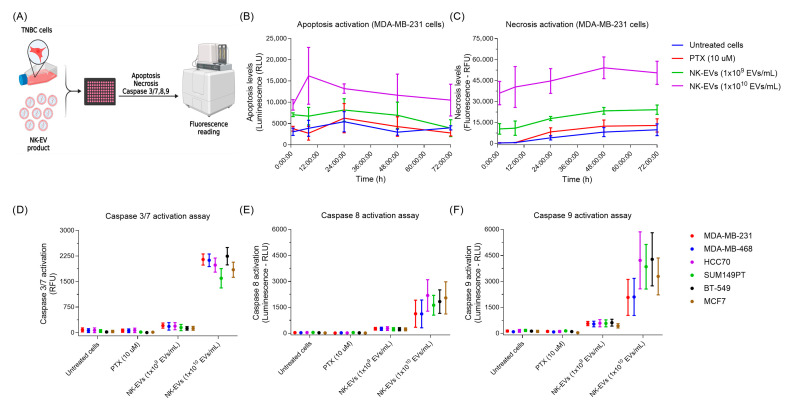
NK-EVs induce cell-death pathways in triple-negative breast cancer cell lines. (**A**) Schematic overview of the in vitro assay design. Various TNBC cell lines were treated with NK-EVs at two concentrations (1 × 10^9^ and 1 × 10^10^ EVs/mL). Paclitaxel (PTX; 10 µM) was included for comparison. (**B**) Representative apoptosis kinetics measured by Annexin V binding (RLU) over time in MDA-MB-231 cells. Data are shown as mean ± SEM from three independent experiments with technical duplicates. (**C**) Representative necrosis kinetics measured by DNA binding (RFU) over time in MDA-MB-231 cells (up to 72 h). Data are presented as mean ± SEM, from three independent experiments with technical duplicates. Caspase activation after 90 min of NK-EV treatment in various TNBC and breast cell lines: (**D**) Caspase 3/7 activity (RFU), (**E**) Caspase 8 activity (RLU), and (**F**) Caspase 9 activity (RLU). Data represent mean ± SEM from four independent experiments, each with technical duplicates.

To elucidate the functional MoA underlying NK-EV-induced cytotoxicity, we performed a series of plate-based assays to examine apoptosis and necrosis in TNBC cell lines treated with NK-EVs (Figure 3A). Treatment with a high concentration of NK-EVs (1 × 10^10^ EVs/mL) led to a rapid and significant increase in both apoptotic and necrotic cell death over 72 h, compared to the untreated and PTX groups, which we previously observed required 48 to 72 h to achieve significant cell killing (Figure 3B,C). Correspondingly, activation of caspase-3/7, -8, and -9 was markedly elevated following 90 min of treatment, indicating fast-acting cytotoxic kinetics (Figure 3D–F). In contrast, a lower NK-EV concentration (1 × 10^9^ EVs/mL) led to a small, non-significant increase in apoptosis and caspase activation, but significantly increased necrosis. Collectively, these findings indicate that NK-EV-mediated killing engages multiple cell death pathways, including both caspase-dependent apoptosis and necrosis.

### 3.4. NK-EVs Induced Potent Apoptosis-Mediated Cytotoxicity in TNBC-PDX Organotypic Slice Cultures

**Figure 4:**
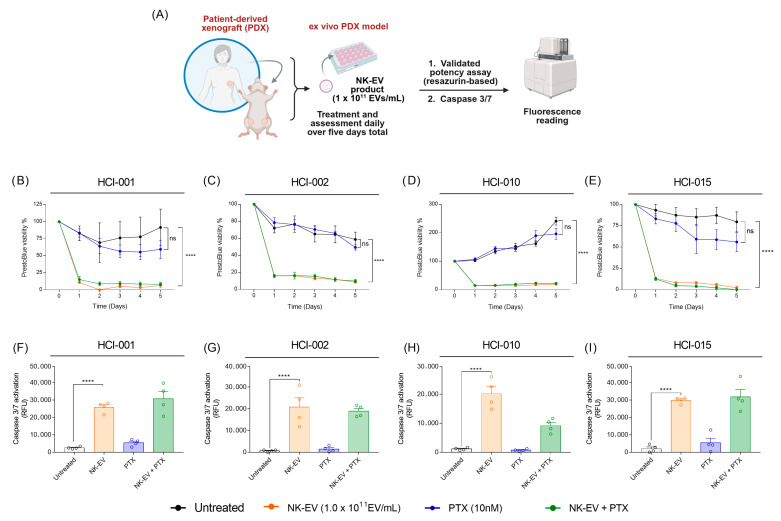
NK-EV treatment induces apoptosis-mediated cell death in human TNBC patient-derived xenograft (PDX) organotypic slice cultures. (**A**) Schematic representation of the cytotoxic co-culture assay. TNBC PDX fragments were treated with NK-EVs daily for five consecutive days with NK-EVs (1.0 × 10^11^ EVs/mL orange), paclitaxel (10 nM PTX; blue), a combination of paclitaxel and NK-EVs (green), or left untreated (black). Cell viability was assessed using a resazurin-based assay. (**B**–**E**) Resazurin-based cell viability measurements in four independent TNBC PDX organotypic slice cultures (i.e., from four individual TNBC patients): (**B**) HCI-001, (**C**) HCI-002, (**D**) HCI-010, and (**E**) HCI-015. Data represent mean ± SEM from five independent experiments. (**F**–**I**) Caspase 3/7 activation (RFU) after 90 min of treatment in the corresponding PDX organotypic slice cultures: (**F**) HCI-001, (**G**) HCI-002, (**H**) HCI-010, and (**I**) HCI-015. Data are shown as mean ± SEM from four independent experiments. Statistical significance is indicated as ****: *p* < 0.0001; ns: nonsignificant.

To further validate these findings in a physiologically relevant three-dimensional model, ex vivo experiments were conducted using organotypic slice cultures derived from four distinct TNBC PDXs (HCI-001, HCI-002, HCI-010, and HCI-015), each originating from a different TNBC patient [36]. To assess cytotoxicity, PDX organotypic slice cultures were treated with a high dose of NK-EVs (1 × 10^11^ EVs/mL), alone or in combination with PTX, every 24 h for a total of 5 days (Figure 4A). For each timepoint, viability was normalized to the day 0 (pre-treatment) baseline. NK-EVs alone or combined with PTX induced significant cytotoxicity across all PDX models within 24 h of treatment (Figure 4B–E). Repeated administration of NK-EVs maintained low cell viability over time, suggesting that NK-EV treatment effectively reduced the viability of heterogeneous tumor populations. In contrast, PTX monotherapy had minimal impact on cell viability relative to the untreated controls. This is consistent with prior PTX exposure of these PDX models (HCI-001, HCI-010, and HCI-015; Table S1) and their associated resistance. PTX resistance in HCI-001 was confirmed in our previous in vivo studies, where the highest dose of PTX tested (10 mg/kg, approximately two-fold higher than clinically relevant human dosing) failed to inhibit PDX tumor growth [40]. There were no significant differences in cell viability observed between NK-EV monotherapy and NK-EV with PTX combinational therapy. Caspase 3/7 activation, a surrogate for apoptosis activation, was then measured after 90 min of NK-EV treatment. Remarkably, NK-EV-treated PDX cultures exhibited a rapid and robust increase in caspase 3/7 activation, whereas untreated and PTX-treated groups showed negligible activation (Figure 4F–I). Notably, the combination of NK-EVs with PTX did not further enhance caspase 3/7 activation relative to NK-EV treatment alone in any PDX model. This finding suggests that NK-EV treatment alone is sufficient to trigger rapid, apoptosis-mediated tumor cell death.

### 3.5. NK-EVs Suppress TNBC Stemness by Disrupting CSC-Associated Gene Expression and Tumorsphere Initiation

**Figure 5:**
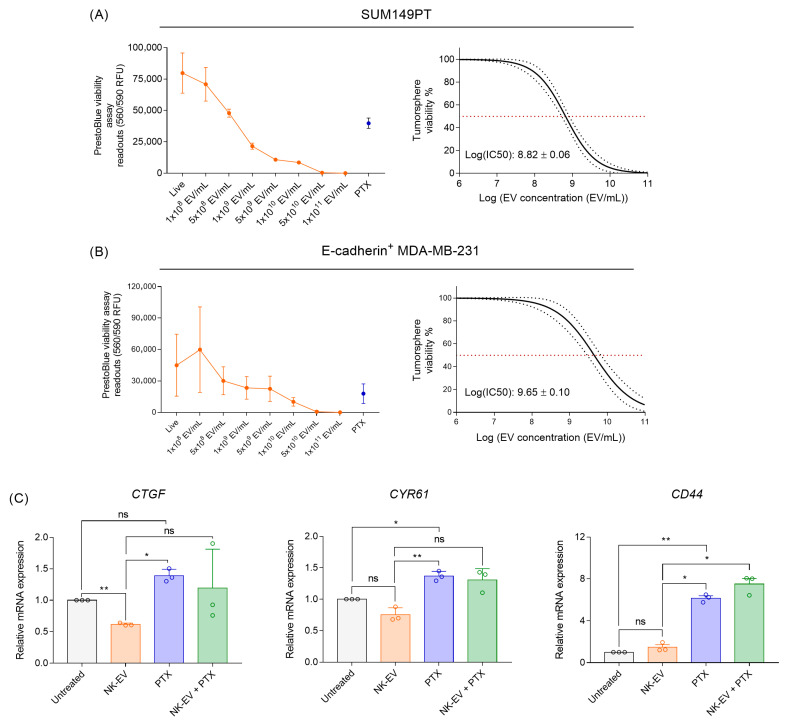
NK-EV treatment disrupts TNBC cancer stem cell-associated phenotypes. The tumorsphere formation assays were conducted on (**A**) SUM149PT (2000 cells) following 7 days of treatment and (**B**) E-cadherin^+^ MDA-MB-231 (1500 cells) following 4 days of treatment with a single dosage of NK-EVs (orange) or 10 nm paclitaxel (PTX; blue). Results are presented as (i) RFU and (ii) EC_50_ curve analysis for NK-EV treatment with 95% confidence interval and prediction bands (red dashed line represents 50% response). (**C**) RT-qPCR analysis of *CTGF*, *CYR61*, and *CD44* gene expression in MDA-MB-231 cells after 72 h of NK-EV treatment. Treatment groups include untreated control (gray), 1.0 × 10^9^ NK-EVs/mL (orange), 10 nM paclitaxel (blue), a combination of 1.0 × 10^9^ EVs/mL NK-EVs and paclitaxel (green). Data are shown as the mean ± SEM from three independent experiments, each with technical duplicates. ns: non-significant, *: *p* < 0.05, **: *p* < 0.01.

While NK-EVs effectively reduced the viability of the bulk tumor cell population, we next investigated their impact on the TNBC CSC subpopulations. To assess tumorsphere formation, epithelial-like TNBC cell lines (SUM149PT and E-cadherin^+^ MDA-MB-231) were cultured under serum-free, non-adherent conditions to enrich cells with CSC-like properties. Cells were treated with NK-EVs for 72 h to allow tumorsphere formation. In both cell lines, NK-EV treatment significantly reduced tumorsphere cell viability in a dose-dependent manner, yielding average log(EC_50_) values of 8.82 ± 0.06 EVs/mL for SUM149PT and 9.65 ± 0.10 EVs/mL for E-cadherin^+^ MDA-MB-231 (Figure 5A,B). To further assess the effect of NK-EVs on the CSC-promoting Hippo-YAP pathway, expression levels of *CTGF*, *CYR61*, and *CD44* were measured after 72 h of treatment. NK-EV treatment alone resulted in the greatest suppression of all evaluated CSC-promoting genes (Figure 5C). Conversely, PTX treatment, known to enrich CSCs, markedly upregulated their expression. The combination of PTX and NK-EV treatment did not fully reverse the PTX-induced gene upregulation. Accordingly, no evidence of synergy was observed under these conditions, suggesting alternative experimental contexts may be required to more accurately assess the therapeutic potential of this combination. Collectively, these results indicate that NK-EV treatment of TNBC cells alone disrupted Hippo-YAP signaling and tumorsphere initiation, key characteristics of the CSC subpopulation.

### 3.6. NK-EVs Induce Early Apoptosis in TNBC CSCs

**Figure 6:**
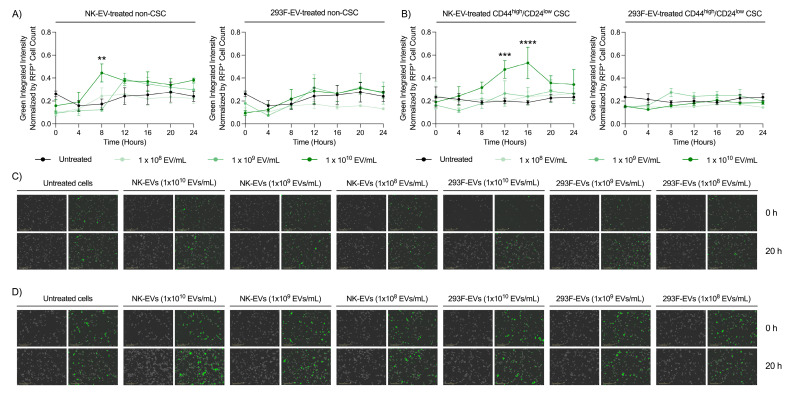
NK-EV treatment rapidly induces cytotoxicity in MDA-MB-231 TNBC cancer stem cells (CSC). RFP^+^ MDA-MB-231 cells were FACS sorted as CD44^high^/CD24^low^ CSCs and non-CSCs. Cells were incubated with green Annexin-V in the Sartorius S3 for live-cell imaging over a 24 h period. Treatment groups included a low (1 × 10^8^ EVs/mL), medium (1 × 10^9^ EVs/mL), or high (1 × 10^10^ EVs/mL) dose of NK-EVs or 293F-EVs (control), along with an untreated control. Green Annexin-V fluorescence intensity was quantified for (**A**) non-CSCs and (**B**) CD44^high^/CD24^low^ CSCs. Representative bright field and green fluorescence images illustrate Annexin-V expression of (**C**) non-CSCs and (**D**) CSCs following 20 h of treatment. Data are shown as the mean ± SEM from four independent experiments, each with technical triplicates. ns: non-significant, **: *p* < 0.01, ***: *p* < 0.001, ****: *p* < 0.0001.

Live cell imaging was conducted to assess TNBC CSC viability following NK-EV treatment. RFP^+^ MDA-MB-231 cells were sorted by FACS into the CD44^high^/CD24^low^ CSC and non-CSC populations. The sorted cell populations were then treated with low (1.0 × 10^8^ EV/mL), medium (1.0 × 10^9^ EV/mL), or high (1.0 × 10^10^ EV/mL) doses of NK-EVs for up to 96 h. The highest NK-EV dose significantly increased Annexin-V expression, a marker of early apoptosis, in both populations within the first 24 h (Figure 6A,B and Figure S3A,B). Peak Annexin-V expression occurred at 8 h in non-CSC and 16 h in CSCs, suggesting a delayed apoptotic response in the CSC subset. Phase contrast and green-channel images validated that the high NK-EV dose elicited the strongest apoptotic response over time (Figure 6C,D and Figure S3E,F). Treatment with the control 293F-EVs did not alter Annexin-V expression or viability in either population (Figure 6A–D), confirming that the observed effects were NK-EV-specific. Collectively, these findings demonstrate that NK-EVs trigger early apoptosis in both TNBC CSCs and non-CSCs, effectively sensitizing these cells to subsequent cell death.

## 4. Discussion

TNBC is the most aggressive breast cancer subtype and remains largely resistant to targeted therapies due to the absence of targetable receptors. Surgery and chemotherapy remain the mainstays of treatment, yet these approaches are limited by off-target toxicity, the development of acquired resistance, and the inadvertent enrichment of CSCs, contributing to tumor relapse and metastasis [3,5,6,7]. To address these challenges, this study evaluated the therapeutic potential of NK-EVs in clinically relevant TNBC models. NK-EVs offer several distinct advantages as anti-cancer agents, including efficient tumor penetration and the capacity to deliver bioactive cargo directly to solid tumors [3,21,41]. As natural derivatives from NK cells, NK-EVs are intrinsically enriched with cytotoxic molecules, endowing them with potent tumor-lytic activity [26,27,28,29]. GMP-grade NK-EVs used in this study were produced from the NK92-MI cell line under feeder-free conditions, as previously characterized by our group [26].

Evaluation of the NK-EVs revealed that their cytotoxicity was predominantly dose-and time-dependent, with significant reductions in cell viability observed as early as 5 h post-treatment. Despite this rapid onset of activity, NK-EV-mediated cytotoxicity peaked within the first 24 h, after which tumor cell proliferation was gradually restored, leading to a recovery of total cell numbers. This transient effect is consistent with previous reports demonstrating that NK-EVs exert a rapid but short-lived cytolytic effect upon interaction with target cells [29,31,42,43,44]. Such behavior may reflect their non-replicative nature and relatively short biological half-life, ranging from a few minutes to approximately 24 h, which facilitates their rapid cellular uptake and systemic clearance [45,46,47]. Notably, long-term NK-EV outcomes reported in the literature are variable, potentially due to differences in NK-EV biomanufacturing, NK cell activation states, and the characteristics of recipient tumor cells [48,49]. The short-term activity observed here may have important therapeutic implications. On one hand, the transient nature of NK-EVs may be advantageous for minimizing the risks associated with sustained immune activation, such as cytokine release syndrome seen in cell-based therapies [21,50]. On the other hand, repeated dosing may be required to maintain durable anti-tumor effects. Importantly, the long-term MoA of NK-EVs may extend beyond direct cytotoxicity. For example, NK-EVs are enriched in immune-regulatory proteins and have been shown to enhance the activity of multiple anti-tumor immune populations (NK cells, CD8+ T cells, and macrophages) [23,51,52,53]. Therefore, their sustained anti-tumor efficacy may be mediated, at least in part, through immunomodulatory effects, warranting further investigations.

To elucidate the manner by which NK-EVs induce cell death in TNBC, a critical aspect for translational development and clinical evaluation, mechanistic assays were implemented to assess interactions with target cells. The two predominant modes of cell death implicated in the NK-EV’s MoA were apoptosis and necrosis, both of which were activated within the first 12 h of treatment. Apoptotic signaling was further examined through caspase activation profiling. During apoptosis, caspases-3/7 serve as key executioner enzymes activated downstream of either caspase-8, which mediates the extrinsic death receptor, or caspase-9, which governs the intrinsic mitochondrial pathway in response to cellular stress. Again, NK-EV treatment resulted in rapid activation of caspases -3/7, -8, and -9. This is consistent with the known proteome of NK-EVs, which contains cytotoxic molecules, including perforin, granzyme A/B, granulysin, and IFNγ, as well as inhibitory miRNAs that mediate targeted killing of malignant cells [26,27,28,29,30]. Furthermore, our group recently reported that the interaction between the NK-EV’s biomolecular corona and its target cancer cells was essential to its cytotoxic functioning [30]. More specifically, the biomolecular corona influenced surface receptor expression, the release of various cytokines, and caspase activation, aspects that are critical for proper NK-EV functioning. Altogether, these findings suggest that the MoA of NK-EVs is largely mediated through their biomolecular composition. Upon interaction with their target cells, the NK-EVs’ composition allows for the activation of multiple caspase-mediated apoptosis pathways, alongside necrosis, to achieve efficient tumor cell elimination.

While cell line-based assays enable rapid evaluation of anticancer therapeutics, their predictive value for clinical outcomes often does not translate to the results obtained during clinical trials [54]. To better capture the complexity of human tumors, preclinical three-dimensional PDX models obtained from patient biopsies are increasingly employed for testing therapeutics, as they preserve the original tumor’s architecture and heterogeneity, closely reflecting patient tumors and treatment response [33,55,56]. To account for disease heterogeneity, various ex vivo TNBC PDX models with unique clinical profiles were tested to validate the NK-EVs’ MoA [36]. Consistent with the results obtained in TNBC cells, NK-EV treatment of all TNBC PDX models rapidly induced caspase 3/7 activation and reduced cell viability by over 80% within the first 24 h of exposure. Together, these findings suggest that NK-EVs exert potent cytotoxic effects in a three-dimensional, heterogeneous TNBC model.

Finally, the impact of NK-EVs on the TNBC CSC subpopulation was investigated. McCune and Kornbluth first demonstrated that NK-EVs are cytolytic toward a subset of treatment-resistant leukemic cells exhibiting CSC-like phenotypes [3]. In line with these findings, this study demonstrated that NK-EVs effectively suppress CSC viability and tumorsphere formation, a functional hallmark of CSCs, in TNBC. Specifically, NK-EV treatment increased Annexin-V expression, an early marker for apoptosis, in both CSC and non-CSC fractions, indicating induction of apoptosis across the entire tumor cell population. However, repeated NK-EV dosing may be necessary to achieve complete cell killing of the CSC populations, similarly to what was previously observed in TNBC cell lines. Mechanistically, NK-EV exposure downregulated the expression of *CTGF*, *CYR61*, and *CD44* genes, key components of the stemness-associated Hippo-YAP pathway. However, it remains unclear whether this effect arises from direct cytotoxicity, or from the delivery of inhibitory proteins or miRNA. Although further mechanistic and in vivo validation is needed, these findings suggest that NK-EVs can target CSCs, potentially reducing the risk of tumor recurrence and metastatic spread. Future studies should explore strategies to enhance NK-EV potency and persistence, including engineering approaches or cargo designed to improve tumor targeting and sustain cytotoxic activity against both bulk tumor cells and CSCs.

## 5. Conclusions

In conclusion, this study underscores NK-EVs as a promising immunotherapeutic platform for TNBC, a difficult-to-treat cancer with limited treatment options. NK-EV treatment alone induced a rapid, dose-dependent cytotoxic effect across TNBC cell lines and clinically relevant PDX organotypic slice cultures through activation of the apoptotic and necrotic pathways. Importantly, NK-EVs also impaired CSC functionality and viability, demonstrating efficacy against the full spectrum of tumor cell populations. Altogether, these findings highlight NK-EVs as a novel and versatile anti-cancer modality with significant potential to suppress tumor growth, limit recurrence, and improve therapeutic outcomes in TNBC.

## Data Availability

The original contributions presented in this study are included in the article/Supplementary Material. Further inquiries can be directed to the corresponding authors.

## Supplementary Materials

The following supporting information can be downloaded at: https://www.mdpi.com/article/10.3390/nano16090525/s1, Figure S1: Repeated NK-EV treatment sensitizes TNBC cells. TNBC cells were pre-treated with a high dose of NK-EVs (5 × 10^10^ EVs/mL). Once cells reached confluency, pre-treated or untreated control cells were incubated for 5 h with various NK-EV concentrations, followed by an endpoint resazurin-based cell viability assay. Results for the TNBC cell line (*i*) MDA-MB-231 and (*ii*) SUM149PT are presented as (A) RFU (black dashed line indicates lysed-cell control using Triton-X), (B) EC_50_ curve fitting for NK-EV treatment with 95% confidence and prediction intervals (red dashed line represents 50% response) and (C) comparison of logEC_50_ values. Data are shown as mean ± SEM in (A) and mean ± SD in (C) from six independent experiments, each with technical triplicates, **** *p* ≤ 0.0001.; Figure S2: Cytotoxic effects of NK92-EVs on TNBC cell lines after 24 h of co-culture. 1500 MDA-MB-231 and SUM149PT cells were seeded and either left untreated (right panel) or co-incubated with a high dose of NK-EVs (1 × 10^11^ EVs/mL; left panel). Images were captured using an EVOS FL microscope with a 4X objective lens at the 24 h-mark.; Figure S3: NK-EV treatment selectively targeted MDA-MB-231 cancer stem cells (CSC) over 96 h. RFP+ MDA-MB-231 cells were FACS-sorted as CD44^high^/CD24^low^ CSCs or non-CSCs and subsequently treated with high (1 × 10^10^ EVs/mL), medium (1 × 10^9^ EVs/mL), or low (1 × 10^8^ EVs/mL) dose of NK-EVs or control 293F-EVs. Cells were incubated with green Annexin-V for 96 h in the Sartorius S3 live cell imaging system. Quantification parameters include (A) RFP signal as a proxy for cell viability of MDA-MB-231 and (B) green Annexin-V expression representative of early apoptosis among NK-EV-treated RFP^+^ MDA-MB-231 cells. (C) RFP expression and (D) Annexin-V expression in 293F-EV-treated controls are shown as controls. Representative brightfield and red fluorescence images illustrate the viability of (E) non-CSCs and (F) CSCs after 96 h. Data are shown as mean ± SEM from four independent experiments with technical triplicates. Statistical differences were assessed using the Wilcoxon test, where ns: non-significant, ****: *p* < 0.0001.; Table S1: Patient information for tested TNBC PDX Legend: IDC, invasive ductal carcinoma. Based on https://pubmed.ncbi.nlm.nih.gov/35221336/ (accessed on 23 April 2026); Table S2: List of primers used for RT-qPCR; Table S3: Human cancer cell lines investigated. Subtype legend: Basal-like, including subtypes BL1 (basal-like 1), BL2 (basal-like 2) and IM (immunomodulatory); (2) Mesenchymal-like, including subtypes M (mesenchymal) and MSL (mesenchymal stem-like); and, (3) LAR (luminal androgen receptor) with an LAR subtype. Histology legend: AC, adenocarcinoma; IBC, inflammatory breast cancer; DC, Ductal carcinoma. Based on: (1) https://www.ncbi.nlm.nih.gov/pmc/articles/PMC3532890/ (accessed on 23 April 2026), and (2) https://www.ncbi.nlm.nih.gov/pmc/articles/PMC3127435/ (accessed on 23 April 2026).

## Abbreviations

The following abbreviations are used in this manuscript:

AO	Acridine Orange
CM	Conditioned Medium
CTLs	Cytotoxic T Cells
CD	Cluster of Differentiation
CSC	Cancer Stem Cell
E: T	Effector: Target
EVs	Extracellular Vesicles
FACS	Fluorescence-Activated Cell Sorting
FBS	Fetal Bovine Serum
FPLC	Fast Protein Liquid Chromatography
MoA	Mode-of-Action
NK	Natural Killer
NK-EVs	Natural Killer Cell-Derived Extracellular Vesicles
PBS	Phosphate-Buffered Saline
PDX	Patient-Derived Xenografts
PI	Propidium Iodide
TNBC	Triple-Negative Breast Cancer
RFU	Relative Fluorescence Units
SD	Standard Deviation
SEC	Size-Exclusion Chromatography
SEM	Standard Error of the Mean
UF	Ultrafiltration
