# Supplementary material for: Natural Killer Cell-Derived Extracellular Vesicles Exhibit Cytotoxicity Against Bulk Tumor Cells and Cancer Stem Cells in Triple-Negative Breast Cancer
Source: Nanomaterials (Basel). 2026 Apr 27;16(9):525. doi: 10.3390/nano16090525 (PMC13165227; doi:10.3390/nano16090525)
Supplement: Supplementary file 1 [file nanomaterials-16-00525-s001.zip › nanomaterials-4232658-supplementary.pdf]

Supplemental Information

Natural Killer Cell-Derived Extracellular Vesicles Exhibit Cytotoxicity Against Bulk Tumor Cells and Cancer Stem Cells in Triple-Negative Breast Cancer

Melanie Kirkby [1,2,†], Frederic St-Denis-Bissonnette [1,2,†], Marena D. Diab [2], Karan Mediratta [2], Anna Korobkow [1,2], James Humber [2], Peter Han [2], Gauri Muradia [1], Michele Ardolino [2,3,4], Seung-Hwan Lee [2,3], Derrick J. Gibbings [5], Dylan Burger [5,6], Lisheng Wang [2,3,7,*] and Jessie R. Lavoie [1,2,*]

[1] Biologic and Radiopharmaceutical Drugs Directorate, Health Canada, Ottawa, ON K1A 0K9, Canada; mkirk071@uottawa.ca (M.K.); fstde005@uottawa.ca (F.S.-D.-B.); akoro009@uottawa.ca (A.K.); gauri.muradia@hc-sc.gc.ca (G.M.)
[2] Department of Biochemistry, Immunology and Microbiology, University of Ottawa, Ottawa, ON K1H 8M5, Canada; mdiab042@uottawa.ca (M.D.D.); kmedi072@uottawa.ca (K.M.); jhumb080@uottawa.ca (J.H.); zhan100@uottawa.ca (P.H.); m.ardolino@uottawa.ca (M.A.); seunglee@uottawa.ca (S.-H.L.)
[3] Centre for Infection, Immunity and Inflammation, University of Ottawa, Ottawa, ON K1H 8M5, Canada
[4] Cancer Therapeutics Program, Ottawa Hospital Research Institute, Ottawa, ON K1H 8L6, Canada
[5] Department of Cellular and Molecular Medicine, University of Ottawa, Ottawa, ON K1H 8M5, Canada; gibbings@uottawa.ca (D.J.G.); dburger@uottawa.ca (D.B.)
[6] Kidney Research Centre, Ottawa Hospital Research Institute, Ottawa, ON K1H 8L6, Canada
[7] Regenerative Medicine Program, Ottawa Hospital Research Institute, Ottawa, ON K1H 8L6, Canada
* Correspondence: lisheng.wang@uottawa.ca (L.W.); jessie.lavoie@hc-sc.gc.ca (J.R.L.)
† These authors contributed equally to this work.

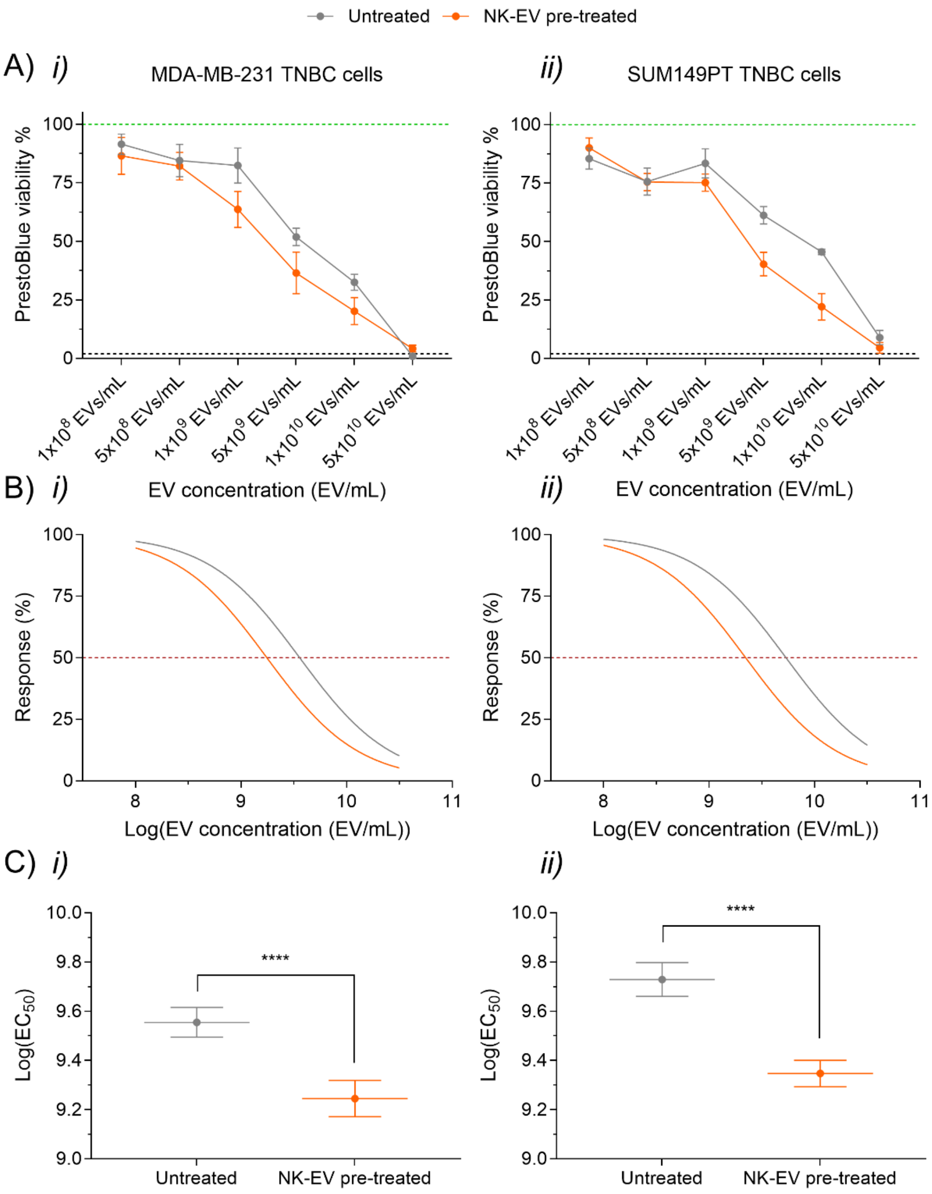

Figure S1. Repeated NK-EV treatment sensitizes TNBC cells. TNBC cells were pre-treated with a high dose of NK-EVs (5×10^{10} EVs/mL). Once cells reached confluency, pre-treated or untreated control cells were incubated for 5 h with various NK-EV concentrations, followed by an endpoint resazurin-based cell viability assay. Results for the TNBC cell line (*i*) MDA-MB-231 and (*ii*) SUM149PT are presented as **(A)** RFU (black dashed line indicates lysed-cell control using Triton-X), **(B)** EC_{50} curve fitting for NK-EV treatment with 95% confidence and prediction intervals (red dashed line represents 50% response) and **(C)** comparison of $logEC_{50}$ values. Data are shown as mean ± SEM in (A) and mean ± SD in (C) from six independent experiments, each with technical triplicates, ****p ≤ 0.0001.

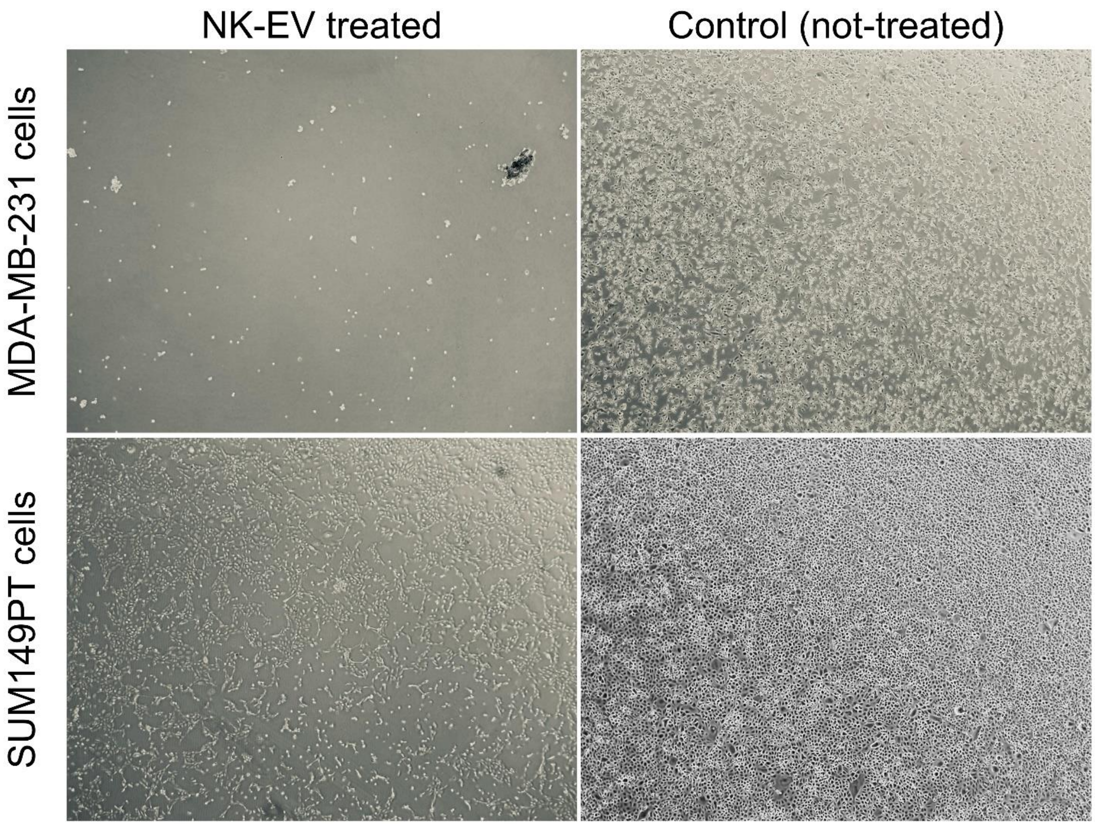

Figure S2. Cytotoxic effects of NK92-EVs on TNBC cell lines after 24 of co-culture. 1500 MDA-MB-231 and SUM149PT cells were seeded and either left untreated (right panel) or co-incubated with a high dose of NK-EVs (1 × 10^{11} EVs/mL; left panel). Images were captured using an EVOS FL microscope with a 4× objective lens at the 24 -mark.

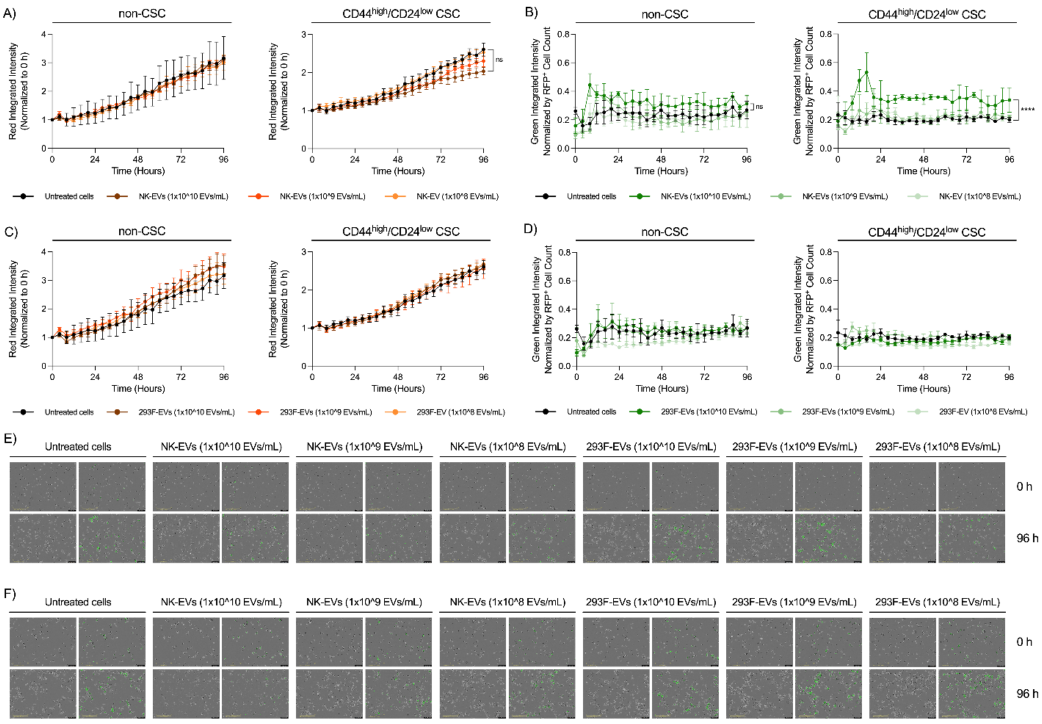

Figure S3. NK-EV treatment selectively targeted MDA-MB-231 TNBC cancer stem cells (CSC) over 96 hours. RFP^+ MDA-MB-231 cells were FACS-sorted as $CD44^{high}/CD24^{low}$ CSCs or non-CSCs and subsequently treated with high (1×10^{10} EVs/mL), medium (1×10^{9} EVs/mL), or low (1×10^{8} EVs/mL) dose of NK-EVs or control 293F-EVs. Cells were incubated with green Annexin-V for 96 h in the Sartorius S3 live cell imaging system. Quantification parameters include **A)** RFP signal as a proxy for cell viability of MDA-MB-231 and **B)** green Annexin-V expression representative of early apoptosis among NK-EV-treated RFP+ MDA-MB-231 cells. **C)** RFP expression and **D)** Annexin-V expression in 293F-EV-treated controls are shown as controls. Representative brightfield and red fluorescence images illustrate the viability of **E)** non-CSCs and **F)** CSCs after 96 hours. Data are shown as mean ± SEM from four independent experiments with technical triplicates. Statistical differences were assessed using the Wilcoxon test, where ns: non-significant, ****: $p < 0.0001$.

Table S1. Patient information for tested TNBC PDX.

Patient ID	Demographic	Collection source	Pre-collection systemic treatment	Clinical metastasis
HCI-001	40-year-old Caucasian; IDC (Stage IV)	Primary breast tumour	Paclitaxel 2007	Lung
HCI-002	61-year-old Caucasian; IDC	Primary breast tumour	None	Bone, lymph node
HCI-010	49-year-old Caucasian; IDC	Pleural effusion	Cyclophosphamide; doxorubicin; paclitaxel 2007; liposomal doxorubicin 2008; zolendronic acid 2008; Capecitibine 2008-2009; docetaxel 2008	Lung
HCI-015	49-year-old Caucasian; IDC	Brain metastasis	Doxorubicin, cyclophosphamide, paclitaxel 2010	Brain

Legend: IDC, invasive ductal carcinoma. Based on https://pubmed.ncbi.nlm.nih.gov/35221336/.

Table S2. List of primers used for RT-qPCR.

Gene	Forward primer sequence	Reverse primer sequence
GAPDH	AATGGGCAGCCGTTAGGAAA	GCGCCCAATACGACCAAATC
CTGF	AGGAGTGGGTGTGTGACGA	CCAGGCAGTTGGCTCTAATC
CYR61	AGCCTCGCATCCTATACAACC	TTCTTTCACAAGGCGGCACTC
CD44	AGACAACCACAAGGATCACTGATG	TCCAGTTTCCTTCATAAGCATGG

Table S3. Human cancer cell lines investigated.

	Breast cancer cell models					
	**MDA-MB-468**	**HCC70**	**SUM149PT**	**BT-549**	**MDA-MB-231**	**MCF7**
**Ethnicity**	Black	Black	?	Caucasian	Caucasian	Caucasian
**Source**	Breast	Breast	Breast	Breast	Breast	Breast
**Subtype**	BL1	BL2	BL2	M	MSL	Luminal A
**Histology**	AC	DC	IBC	DC	AC	AC
**ER**	Neg	Neg	Neg	Neg	Neg	Pos
**PR**	Neg	Neg	Neg	Neg	Neg	Pos
**HER2**	Neg	Neg	Neg	Neg	Neg	Neg
**EGFR**	Very high	High	Medium	Medium	Medium	Neg

Subtype legend: Basal-like, including subtypes BL1 (basal-like 1), BL2 (basal-like 2) and IM (immunomodulatory); (2) Mesenchymal-like, including subtypes M (mesenchymal) and MSL (mesenchymal stem-like); and, (3) LAR (luminal androgen receptor) with an LAR subtype. Histology legend: AC, adenocarcinoma; IBC, inflammatory breast cancer; DC, Ductal carcinoma. Based on: 1) https://www.ncbi.nlm.nih.gov/pmc/articles/PMC3532890/, and 2) https://www.ncbi.nlm.nih.gov/pmc/articles/PMC3127435/.
